# Development of an Indoor Location Based Service Test Bed and Geographic Information System with a Wireless Sensor Network

**DOI:** 10.3390/s100402957

**Published:** 2010-03-30

**Authors:** Shau-Shiun Jan, Li-Ta Hsu, Wen-Ming Tsai

**Affiliations:** Department of Aeronautics and Astronautics, National Cheng Kung University, Tainan 70101, Taiwan; E-Mails: qmohsu@gmail.com (L.-T.H.); darsetsai@yahoo.com.tw (W.-M.T.)

**Keywords:** wireless sensor network, indoor positioning system, geographic information system, location based service

## Abstract

In order to provide the seamless navigation and positioning services for indoor environments, an indoor location based service (LBS) test bed is developed to integrate the indoor positioning system and the indoor three-dimensional (3D) geographic information system (GIS). A wireless sensor network (WSN) is used in the developed indoor positioning system. Considering the power consumption, in this paper the ZigBee radio is used as the wireless protocol, and the received signal strength (RSS) fingerprinting positioning method is applied as the primary indoor positioning algorithm. The matching processes of the user location include the nearest neighbor (NN) algorithm, the K-weighted nearest neighbors (KWNN) algorithm, and the probabilistic approach. To enhance the positioning accuracy for the dynamic user, the particle filter is used to improve the positioning performance. As part of this research, a 3D indoor GIS is developed to be used with the indoor positioning system. This involved using the computer-aided design (CAD) software and the virtual reality markup language (VRML) to implement a prototype indoor LBS test bed. Thus, a rapid and practical procedure for constructing a 3D indoor GIS is proposed, and this GIS is easy to update and maintenance for users. The building of the Department of Aeronautics and Astronautics at National Cheng Kung University in Taiwan is used as an example to assess the performance of various algorithms for the indoor positioning system.

## 1. Introduction

The location based service (LBS) subscriber base is forecasted to reach 680 million people worldwide, because LBS can be applied to many fields, such as local entertainment services, personal security applications and local traffic navigation systems [1]. Importantly, LBS can combine the positioning tags with the local information, and it has been applied to the scientific fields as well [2]. Currently, many smart phones use global positioning system (GPS) to provide navigation and positioning services. However, due to the propagation limit of the GPS signals, these services are available only while the user is in the outdoors. To provide the seamless navigation and positioning services for indoor environments, the wireless sensor network (WSN) might be a solution for this case. Due to the mobility and low cost of WSN, the sensors can be deployed inside buildings. Each sensor acts as a radio beacon to provide positioning services in the indoor environments. Several researches have proposed methods for realizing the indoor positioning system by the WSN [3–5]. Since the signal strength decays as the signal propagation distance increases [6], one can use this propagation characteristic to estimate the user location. The main concept is to use the received signal strength (RSS) as the tag of the location. For instance, the most common method is the triangulation method [7] which uses the propagation model to calculate the signal transmission distance between the transmitter and the receiver, and then estimates the user location by the quadratic iterative least square (QILS) method [8]. However, the positioning accuracy of the triangulation method is usually not sufficient to meet the requirements of indoor applications. Another method is the fingerprinting method [9]. In general, this method can obtain more accurate positioning results. To implement the fingerprinting method, two stages are required, namely the calibration stage and the positioning stage. The calibration stage is the data collection and the buildup of the database for positioning. In the positioning stage, one uses the matching algorithm to find the most likely user location. Ni *et al*. used radio frequency identification (RFID) technique to develop the LANDMARC indoor location sensing system in [4], and they also pointed out that the matching algorithm, the nearest neighbor (NN) method, may get better positioning solution by selecting more reference points in an indoor positioning system. According to their experimental results, selecting four reference points could get more desirable results. However, the indoor environment is complicated, for example, the arrangement of the furniture and the movements of people might block the signal or cause severe attenuation of the signal strength. In addition, the uncertainties of the indoor environment might affect the positioning results. To enhance the positioning performance, Wang *et al*. [10] combined the Kalman filter and the particle filter with the indoor positioning system and analyzed their positioning performance. Wang *et al*. [10] also suggested that the indoor environment is not a linear model, so although the Kalman filter is commonly used in the object tracking applications, it is not suitable for the indoor positioning system. An alternative filter approach, the particle filter might be a better option for the nonlinear and non-Gaussian indoor environments [10]. In addition, since the received signal strength might not be the same even when the user is standing at the same location. To overcome this problem, Roos *et al.* proposed the probabilistic approach to handle the fluctuation phenomenon of the signal strength in [11], namely the kernel method and the histogram method. According to the experimental results in [11], the probabilistic approach has better positioning performance than the use of the NN algorithm.

In light of the above discussion, the fingerprinting method is applied to implement the indoor positioning system. The matching algorithms used in this paper are the NN method, the k-weighted nearest neighbors (KWNN) method [12] and the probabilistic approach [11]. We then compare the positioning results under various architectures. In addition, the particle filter [13] is used in this paper to enhance the performance of the developed indoor positioning system. Most of the current researches focus on the analysis of the indoor positioning algorithms and very few researches show how to build a complete indoor positioning system. Thus, a rapid and practical procedure is proposed for the construction of a three-dimensional (3D) geographic information system (GIS) based on the computer-aided design (CAD) software and the virtual reality markup language (VRML) technique [14]. The main contributions of this paper are the development of a low cost 3D GIS as a LBS test bed and its integration with the indoor positioning system using WSN. The building of the Department of Aeronautics and Astronautics (DAA) at National Cheng Kung University (NCKU) in Taiwan is used as an example to demonstrate the developed system.

Accordingly, the remainder of this paper is organized as follows. In Section 2, we describe the indoor positioning algorithms used in this work. The development procedures of an indoor 3D GIS will be explained in Section 3. In Section 4, several experiments are conducted to evaluate the indoor positioning performance under different architectures. A prototype indoor LBS test bed will be illustrated in this section as well. Finally, Section 5 presents the summary and concluding remarks.

## 2. Indoor Positioning Algorithms

**Figure 1.**
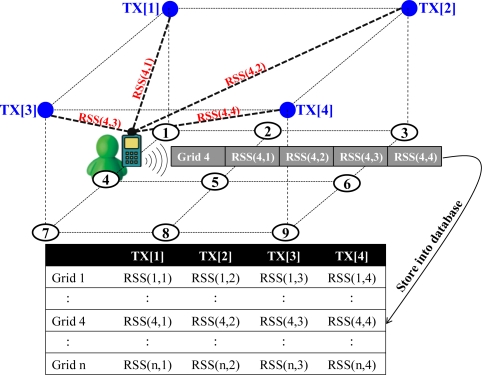
The calibration stage of the fingerprinting algorithm.

Many algorithms such as the triangulation method and the fingerprinting method have been proposed to investigate the possibility for indoor positioning [4,7,12]. The triangulation method requires a signal propagation model to convert RSS values into signal transmission distances, the parameters of the propagation model are strongly location dependent and are different for different environments. Since the indoor environments are extremely complicated, it would be very difficult to find an exact model to describe the relationship between the signal strength attenuation and the transmission distance. In general, a simplified signal propagation model is commonly utilized to calculate the transmission distance [6], and then the QILS method is used to estimate the user location [8]. However, since the multipath effect is severe in indoor environments, the positioning accuracy of the triangulation method is not sufficient in most cases. To overcome this problem, the fingerprinting method was proposed, and the main concept of the fingerprinting method is to use the received signal strength as the tag of the location. It requires two stages to perform the fingerprinting, including the calibration (training) stage and the verification (positioning) stage [9]. In the calibration stage, one assigns an appropriate number of calibration points in the space of interest, and the RSS values of each calibration point have to be measured in sequence and these RSS values are then recorded as the fingerprints in the database. As illustrated in Figure 1, a user with the receiver (RX) visits all the calibration points sequentially to measure the corresponding RSS values of the incoming signals from four transmitters (TXs). For example, when the user visits the calibration point 4, the received RSS values of the incoming signals from four transmitters could be expressed as a vector, and these RSS values and the associated coordinates of the calibration points will be stored into the database for future use.

**Figure 2.**
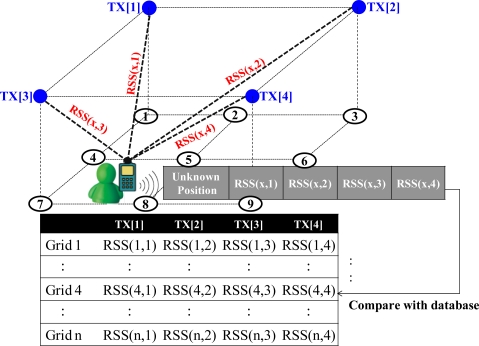
The verification stage of the fingerprinting algorithm.

In the verification stage, if a user obtains the RSS values of the incoming signals from the transmitters, these RSS values might be considered as the tags of the current location, and these RSS measurements will be used in the matching processing to compare with the prerecorded database built in the calibration stage. For instance, a user stays at one unknown location, and this user uses a mobile device to measure RSS values of the incoming signals. The received RSS values are expressed as a vector, and this RSS vector is compared with the prerecorded data. Through the matching process, the most similar set in the database will be admitted as the user’s current location. This verification process is illustrated in Figure 2.

There are many matching algorithms used in the fingerprinting method to estimate the user position, including the nearest neighbor (NN) algorithm [15], the K-weighted nearest neighbors (KWNN) algorithm [9,12], and the probabilistic approach [11]. In this paper all these matching algorithms are implemented to evaluate their positioning performance. Additionally, the particle filter [13] is utilized in this work to gain possible improvement on the positioning performance.

### 2.1. The Nearest Neighbor Algorithm

The main concept of the NN algorithm is to calculate the minimum distance between the measurement and the prerecorded data in the database, and this distance is calculated by Equation (1):

(1) $$d_{j}=\frac{1}{N_{s}}{\left(\sum_{i=1}^{N_{s}}{\left|\mathrm{rss}\left(\mathrm{pos},i\right)-\mathrm{RSS}\left(j,i\right)\right|}^{p}\right)}^{\frac{1}{p}}$$

where *rss*(*pos*,*i*) is the received signal strength of the *i_th_* transmitter at the user position of *pos*, *RSS*(*j*,*i*) is the signal strength of the *i_th_* transmitter measured at the grid point *j* in the database, and *N_s_* is the total numbers of the transmitters. The value of *p* can be chosen as 1 or 2. If *p* equals 1, the generalized distance is called Manhattan distance; otherwise, the distance is called Euclidean distance [15]. Through this calculation, the grid point with the minimum distance is declared as the user position.

### 2.2. The K Weighted Nearest Neighbors Algorithm

Since the NN algorithm determines the user position according to the grid point with minimum distance, if the received signal strength is affected by unexpected factors, such as human motions, the NN algorithm might obtain wrong estimations. To enhance the reliability of indoor positioning, the KWNN algorithm selects more grid points in the database to average the estimation results, and each selected grid point has the specific weighting value according to the distance computed by Equation (1). The grid point with smaller distance has larger weighting value; in contrast, the grid point with larger distance has smaller weighting value. The determination of the weighting value is given by Equation (2):

(2) $$W_{j}=\frac{1/d_{j}}{\sum_{j=1}^{K}1/d_{j}}$$

where *d_j_* is the distance of the *j^th^* grid point, and *K* is the total number of the selected grid points. The estimation of the user position is calculated by Equation (3):

(3) $$\left(X,Y\right)=\sum_{j=1}^{K}\left(W_{j}\times \left(X_{j},Y_{j}\right)\right)$$

where (*X_j_*,*Y_j_*) is the coordinate of *j^th^* selected grid point in X and Y directions, respectively. If the value *K* equals 1, the KWNN algorithm is identical to the NN algorithm.

### 2.3. The Probabilistic Approach

**Figure 3.**
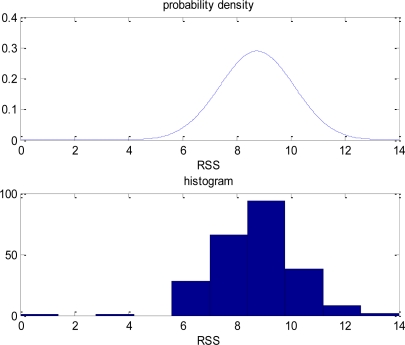
The distribution of the received signal strength at a fixed location.

The received signal strength might not be the same even if the user stands at the same location. Figure 3 gives an example of the distribution of the received signal strength. Figure 3 is the histogram of the received signal strength of the incoming signal from a specific transmitter as one stays statically at the same location. One notes that the received signal strength is not constant even at the fixed location. In Figure 3, the distribution of the received signal strength could be approximated as a Gaussian distribution.

To reduce the computation load, a model is used to describe the distribution of the received signal strength, and the most common model is the Gaussian model which is also known as the kernel method [11]. The kernel method assumes that the received signal strength scatters as a normal distribution. In addition, the received signal strengths from different transmitters are independent. The probability density function of the received signal strength can be expressed as Equation (4):

(4) $$p\left({\mathrm{rss}}_{i},\mathrm{pos}\right)=\frac{1}{\sqrt{2\pi }\sigma_{{\mathrm{rss}}_{i}}}\text{exp}\left(-\frac{{\left({\mathrm{rss}}_{i}-\mu_{{\mathrm{rss}}_{i}}\right)}^{2}}{2\sigma_{{\mathrm{rss}}_{i}}^{2}}\right)$$

where *pos* is the coordinate of the grid point, *rss_i_* is the RSS value of the *i_th_* transmitter at the location *pos*, *μ_rss_* is the mean of the *rss*, and $\sigma_{{\mathrm{rss}}_{i}}^{2}$ is the variance of the *rss*. If there are *M* transmitters, the probability density functions of RSS values from these transmitters can be obtained accordingly. After computing these probability density functions, we could multiply these probability density functions to obtain the likelihood function, as shown in Equation (5):

(5) $$L\left({\mathrm{rss}}_{1},{\mathrm{rss}}_{2}...,{\mathrm{rss}}_{M};\mathrm{pos}\right)=\prod_{i=1}^{M}p\left({\mathrm{rss}}_{i};\mathrm{pos}\right)$$

If a new RSS value set is obtained and there are *M* prerecorded data, the new measurement will be substituted into Equation (5) to get the likelihood function and compare it with the *M* prerecorded data in sequence. If the maximum likelihood is obtained at position pos*, then the position pos* would be declared as the user location.

### 2.4. The Particle Filter

**Figure 4.**
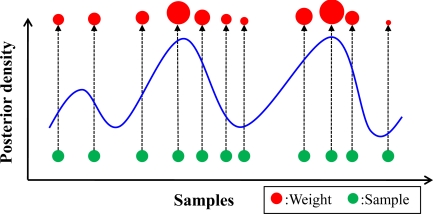
The approximation of the posterior density by the random samplings.

Because the indoor environment is considered as a nonlinear system, the positioning accuracy may be easily affected by several uncontrollable and unexpected factors. In addition, the positioning results based on the matching algorithms might not be desirable due to the signal strength fluctuations. If the user is in motion, it would be difficult to obtain the position estimations which could be approximated to the user indoor trajectories. To improve the positioning accuracy and smoothing the indoor positioning trajectories, an appropriate filtering algorithm is needed. The Kalman filter is a general approach for the tracking applications, but the Kalman filter is suitable for a linear system, and it is not a good option for the indoor positioning system. Considering the complexity of the indoor environment, the particle filter is used in this paper. A particle filter is suitable for a nonlinear and non-Gaussian system, the main algorithm of the particle filter is to implement the recursive Bayesian estimation by the Monte Carlo method, and it represents the posterior density function by a set of random samplings [13,16], as shown in Figure 4.

In Figure 4, each sample is associated with a weight according to the posterior density. If the number of the sample is large, the posterior density can be approximated by Equation (6) [13,16]:

(6) $$p\left(X_{0:k}|Z_{1:k}\right)\approx \sum_{i=1}^{N}w_{k}^{i}\delta \left(X_{0:k}-X_{0:k}^{i}\right)$$

where *X^i^*_0:*k*_ is the set of the random samples at time *k* for *i_th_* sampling point, *X*_0:*k*_ is the set of the system states, *Z*_1:*k*_ is the set of the measurements, *N* is total number of the sampling points, and *w^i^_k_* is the weight of the *i_th_* sampling point at time *k*. The weight update to implement the recursive Bayesian estimations is illustrated as Equation (7) [13,16]:

(7) $$w_{k}^{i}=w_{k-1}^{i}\frac{p\left(Z_{k}|X_{k}^{i}\right)p\left(X_{k}^{i}|X_{k-1}^{i}\right)}{q\left(X_{k}^{i}|X_{0:k-1}^{i},Z_{1:k}\right)}$$

where $q\left(X_{k}^{i}|X_{0:k-1}^{i},Z_{1:k}\right)$ is the importance density. The choice of the importance density has a significant effect on the estimation. The general form of the particle filter is the sequential importance sampling (SIS) particle filter. Assume that the system states evolution is a Markov process, and the current measurement is independent of the previous ones. Then Equations (6) and (7) are simplified to Equations (8) and (9), respectively:

(8) $$p\left(X_{k}|Z_{1:k}\right)\approx \sum_{i=1}^{N}w_{k}^{i}\delta \left(X_{k}-X_{k}^{i}\right)$$

(9) $$w_{k}^{i}=w_{k-1}^{i}\frac{p\left(Z_{k}|X_{k}^{i}\right)p\left(X_{k}^{i}|X_{k-1}^{i}\right)}{q\left(X_{k}^{i}|X_{k-1}^{i},Z_{k}\right)}$$

If ${\left\{X_{k-1}^{i},w_{k-1}^{i}\right\}}_{i=1}^{N}$ and the measurement are available at time *k*, then the ${\left\{X_{k}^{i}\right\}}_{i=1}^{N}$ can be drawn from the importance density, and the weight update is obtained by Equation (9).

However, the SIS particle filter has severe degeneracy [13,16]. A good selection of the importance density or the resampling algorithm might be able to avoid this phenomenon. In general, an appropriate importance density to the real condition is hardly available; therefore, the resampling algorithm is more preferable to the prior approach. The basic concept of the resampling algorithm is to generate a new set of ${\left\{{\tilde{X}}_{k}^{i}\right\}}_{i=1}^{N}$ which eliminates samples with low weights and concentrates on samples with high weights.

Since the optimal importance density is hard to obtain, the prior distribution $p\left(X_{k}|X_{k-1}^{i}\right)$ is often chosen as the importance density, as shown in Equation (10), and the weight update is simplified to Equation (11):

(10) $$q\left(X_{k}^{i}|X_{k-1}^{i},Z_{k}\right)=p\left(X_{k}|X_{k-1}^{i}\right)$$

(11) $$w_{k}^{i}=w_{k-1}^{i}p\left(Z_{k}|X_{k}^{i}\right)$$

**Figure 5.**
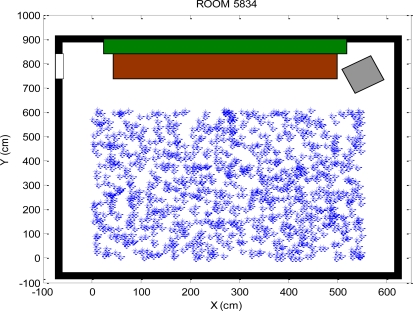
1,000 particles with uniform distribution in the map.

The SIS particle filter with the resampling process is known as the sampling importance resampling (SIR) particle filter [13]. In this paper the SIR particle filter is used to enhance the positioning performance. The procedures of implementing the SIR particle filter are as follows [10,13,16,17]:

Step 1: In the initial stage, *N* random sampling points (particles) is generated at the initial time as ${\left\{X_{0}^{i}\right\}}_{i=1}^{N}$, and each particle is associated with the same weight. This paper generates 1000 particles with the equal weights uniformly distributed in the map, as shown in Figure 5.
Step 2: The evolution of the system states by drawing the ${\left\{X_{k}^{i}\right\}}_{i=1}^{N}$ from $p\left(X_{k}|X_{k-1}^{i}\right)$, if the system model can be expressed as *X_k_* = *f* (*X*_*k*−1_, *v*_*k*−1_), then ${\left\{X_{k}^{i}=f\left(X_{k-1}^{i},v_{k-1}\right)\right\}}_{i=1}^{N}$. To simplify the estimation, the evolution of the system states through the system model is illustrated as Equation (12).
  (12) $$\left[\begin{array}{l} x_{k}^{i}\\ y_{k}^{i} \end{array}\right]=\left[\begin{array}{l} 1\;0\\ 0\;1 \end{array}\right]\left[\begin{array}{l} x_{k-1}^{i}\\ y_{k-1}^{i} \end{array}\right]+\left[\begin{array}{l} \Delta t\;0\\ 0\;\Delta t \end{array}\right]\left[\begin{array}{l} V_{x,k-1}\\ V_{y,k-1} \end{array}\right]+\left[\begin{array}{l} \frac{\Delta t^{2}}{2}\;0\\ 0\;\frac{\Delta t^{2}}{2} \end{array}\right]\left[\begin{array}{l} a_{x,k-1}\\ a_{y,k-1} \end{array}\right]$$
  where *x* and *y* are the horizontal and vertical position coordinates, respectively, *V_x_* and *V_y_* are the associated velocities, *a_x_* and *a_y_* are the associated accelerations, and Δ*t* is the time step.
Step 3: Implement the process of weight update by Equation (11) when the current measurements are available, and assume that the distribution of the likelihood function $p\left(Z_{k}|X_{k}^{i}\right)$ is a Gaussian distribution. The $w_{k-1}^{i}$ is 1/*N* at each time step. Then the weight update is calculated by Equation (13):
  (13) $$w_{k}^{i}=\frac{1}{\sqrt{2\pi }\sigma }\text{exp}\left(-\frac{{\left(x_{k}^{i}-x_{k,\mathrm{KWNN}}\right)}^{2}+{\left(y_{k}^{i}-y_{k,\mathrm{KWNN}}\right)}^{2}}{2\sigma^{2}}\right)$$
  where the *x_k,KWNN_* and *y_k,KWNN_* are the positions computed by the KWNN algorithm, and σ is the standard deviation of the positioning error of the KWNN algorithm. The weight is normalized by Equation (14):
  (14) $${\tilde{w}}_{k}^{i}=\frac{w_{k}^{i}}{\sum_{i=1}^{N}w_{k}^{i}}$$
Step 4: To avoid the degeneracy, the resampling process is applied by generating a new set of ${\left\{{\tilde{X}}_{k}^{i}\right\}}_{i=1}^{N}$ with probability of $\text{Pr}\left({\tilde{X}}_{k}^{i}=X_{k}^{j}\right)={\tilde{w}}_{k}^{j}$.
Step 5: The states estimation at time *k* is the expectation value of the regenerated particles in Step 4 (*i.e.*, ${\left\{{\tilde{X}}_{k}^{i}\right\}}_{i=1}^{N}$).
  (15) $${\hat{X}}_{k}=E\left({\left\{{\tilde{X}}_{k}^{i}\right\}}_{i=1}^{N}\right)$$

**Figure 6.**
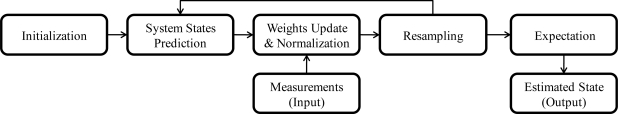
The flowchart of the SIR particle filter.

The flowchart of the SIR particle filter is summarized in Figure 6.

## 3. Geographic Information System

**Figure 7.**
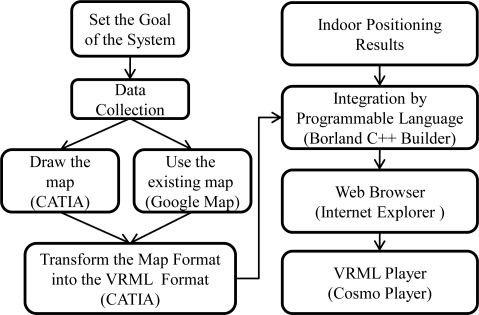
The proposed developmental procedure of the indoor GIS.

There are many smart phones and personal digital assistants (PDAs) which integrate the navigation services with the commercial GIS to provide useful location based information, such as the tourist attractions within a user’s vicinity. Nevertheless, the commercial GIS like the Google map does not provide detail information inside buildings. To achieve a complete LBS, an indoor GIS is needed. The major challenges for constructing an indoor GIS are to establish the indoor GIS and combine it with an indoor positioning system. In general, the program development tool (e.g., Visual C++) and the computer graphic technique (e.g., OpenGL) are used to develop the graphic system. However, if the requirements of the graphic system are complicated, it would be difficult to construct the graphic system by the method mentioned above. In addition, the maintenance and update of this graphic system would be difficult as well. To overcome this problem, the computer-aided design (CAD) software, such as the AutoCAD and CATIA, would be a better solution for the rapid graphic development. With the assistance of the CAD software, the developer could reduce the development time and the workload. However, the graphic format is different for different CAD software, and the compatibility for different graphic format needs to be taken into account. For that reason, the VRML graphic standard is used in this paper. In this paper a solution is proposed to establish the indoor GIS by the CAD software and the VRML technique [14], and then integrate it with the indoor positioning system. The development procedures are as follows: the first step is to set the goal of the GIS. After the setup of the goal, the next step is to collect the information required for this GIS, for instance, the building sizes and the location of the furniture in the room. The third step is to draw the maps by the CAD software. In order to integrate this developed GIS with other applications, the format of the map has to be change to fit the standards of the VRML. Finally, we can use the program development tool (e.g., C++ Builder) to integrate the developed GIS with the indoor positioning system developed in the previous section. The 3D map is shown through the web browser (e.g., Internet Explorer) with the VRML player (e.g., Cosmo Player). The overall development procedure is depicted in Figure 7.

A 3D GIS of the Department of Aeronautics and Astronautics (DAA) building of National Cheng Kung University (NCKU) is developed as an example for demonstration. In this paper the CATIA is used to draw the 3D map of the DAA building and changes the file format of the map to meet the standards of the VRML. The VRML is a 3D graphic display technique used in the Internet. The VRML has high flexibility to combine with other programming languages. For instance, the developer can use C++ or Java to control the object in the VRML [14].

**Figure 8.**
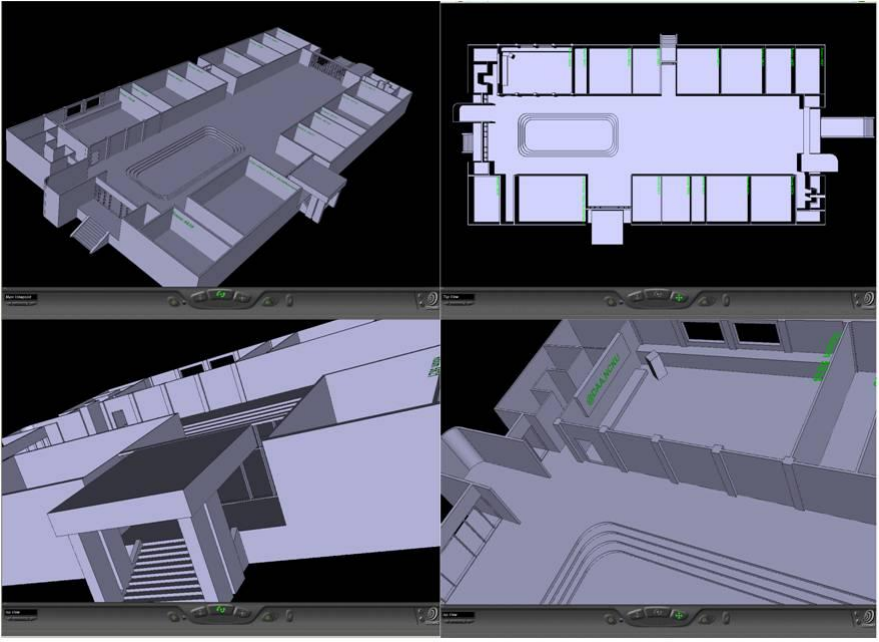
Snapshots of the 3D GIS of the Department of Aeronautics and Astronautics building of National Cheng Kung University.

In this paper, we use C++ Builder to integrate the GIS with the indoor positioning system. The display of the VRML files needs a specific viewer, and the VRML viewer used in this paper is the Cosmo player. Several snapshots of the 3D GIS of the DAA building are shown in Figure 8.

## 4. Experiment Results and Analyses

**Figure 9.**
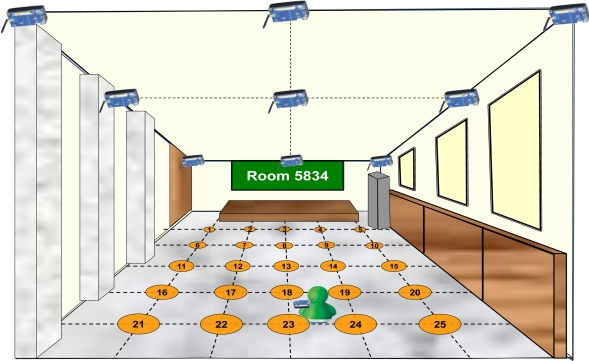
The arrangements of the equipments and the calibration of the grid points.

Considering the power consumption, ZigBee radio was employed to construct the WSN for the indoor positioning test bed. The wireless modules used in this paper is the IRIS® which are developed by Crossbow Inc. The IRIS wireless module can be treated as the transmitter or the receiver. The received data is transmitted *via* USB port. The classroom 5834 of the DAA of NCKU is used as the experiment place. To implement the indoor positioning system, the collection of the database for the positioning system is required. We attach 9 IRIS motes on the ceiling as the transmitter and divide the floor of the classroom 5834 into 25 grid points, and then visit these grid points in sequence to measure the RSS values of the incoming signals from the transmitters which are attached on the ceiling. These RSS values and the corresponding coordinates are saved in the database. The size of each grid is 137.25 cm by 152.5 cm, and the separation distance between each sensor attached on the ceiling is 274.5 cm. The setup and the arrangement of the experiment are shown in Figure 9. All the training samples and the test samples are acquired at the same day. The total training samples are 47,088 samples, and the average training samples at each grid point are 1,883 samples.

**Figure 10.**
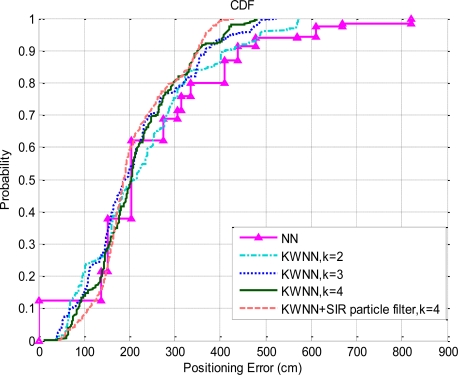
The CDFs of the positioning errors by various matching algorithms.

After the database is built, this paper applies the NN algorithm, the KWNN algorithm and the probabilistic approach based on the kernel method to compare their positioning performance. In the static experiment, we visit all the grid points sequentially to conduct the positioning test, the cumulative distribution functions (CDFs) of the positioning errors with above algorithms are illustrated in Figure 10. The number of the test samples is 13,406 samples.

In Figure 10, the NN algorithm has a 12% probability to make an exact estimation; however, the maximum error of this algorithm is about 8.3 meters. If the indoor positioning system has higher accuracy requirement, then the NN algorithm would not be an appropriate option. In contrast, although the KWNN algorithm cannot make an exact estimation, the maximum error of the KWNN is less than that of the NN algorithm.

**Figure 11.**
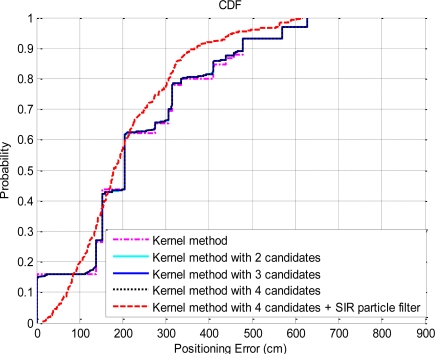
The CDFs of the positioning errors by the kernel method.

When the value of *K* is set to 2, the maximum error is about 5.7 meters. As shown in the figure, the maximum error is reduced as the value of *K* increases, but the improvement diminishes as the value of *K* increases. Ni [4] and Wang [12] suggested that the *K* = 3 or 4 yielded the best positioning results. Our experiment results are in accordance with the conclusions in [4] and [12]. We choose *K* = 4 case to combine with the SIR particle filter; the estimations obtained by the KWNN algorithm are used as the input measurements of the SIR particle filter; the number of particles used in this experiment is 1,000. The velocities and accelerations used in the SIR particle filter for the static tests shown in Figures 10 and 11 are generated by the normal distribution with zero mean, *N*(0,*var*), where *var* is the variance. The velocities (*v*) and the accelerations (*a*) are determined by Equations (16) and (17), respectively. The values of the standard deviation used in Equations (16) and (17) are determined by the empirical data:

(16) v∼N(0 cm/s,250)

(17) $$a∼N\left(0\;\mathrm{cm}/s^{2},20\right)$$

Based on the experimental results, the SIR particle filter significantly improves the positioning accuracy, and the maximum positioning error is reduced to 4.3 meters (in Figure 10). In addition, we apply the probabilistic approach based on the kernel method to analyze the CDFs of the positioning errors. The CDFs of the positioning errors with the probabilistic approach based on the kernel method are shown in Figure 11.

**Figure 12.**
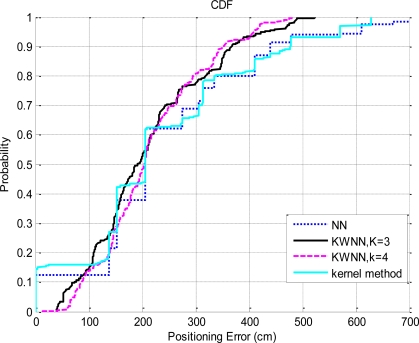
The CDFs of the positioning errors by various matching algorithms.

As shown in Figure 11, the exact estimations are about 15% by applying the probabilistic approach based on the kernel method, and the maximum error is about 6.3 meter. From the experiment results, the probabilistic approach based on the kernel method achieves better positioning accuracy than the use of the NN algorithm alone. In addition, we use the concept similar to the KWNN algorithm to select more candidates and do the average according to the probability of each candidate. Based on the experiment results shown in Figure 11, there is no improvement by selecting more candidates. If we combine the probabilistic approach and the SIR particle filter, the positioning result is similar to the use of the KWNN algorithm. In this case, the estimations obtained by the probabilistic approach are used as the input measurements of the SIR particle filter. Additionally, we compare the positioning performances of the NN algorithm, the KWNN algorithm with parameter *K* = 3 and 4, and the kernel method, and the CDFs of the positioning errors with these methods are shown in Figure 12.

Based on the experiment results shown in Figure 12, although the positioning performance of the kernel method is slightly better than that of the NN algorithm, the KWNN algorithm with *K* = 3 or 4 would still be a better option than the use of the probabilistic approach based on the kernel method.

In this paper a dynamic experiment is also conducted where the user moves from grid point 4 to grid point 24 in a straight line (in Figure 9). We apply the KWNN algorithm with *K* = 1, 2, 3, and 4, and the probabilistic approach based on the kernel method to evaluate their positioning results. Additionally, the SIR particle filter is utilized to gain possible improvement on positioning performance. The velocities and accelerations used in the SIR particle filter are generated by the normal distribution, *N* (*μ*, *var*), where *μ* is the mean and *var* is the variance. The velocities (*v*) and the accelerations (*a*) are determined by Equation (18) and Equation (19), respectively:

(18) v∼N(35 cm/s,250)

(19) $$a∼N\left(0\;\mathrm{cm}/s^{2},20\right)$$

The values of mean and the standard deviation used in Equations (18) and (19) are determined by the empirical data, and these velocities and accelerations can be changes if there are available velocities and accelerations measurements. Table 1 shows the standard deviations of the positioning errors by applying the KWNN algorithm and the KWNN algorithm with the SIR particle filter for different values of *K*.

**Figure 13.**
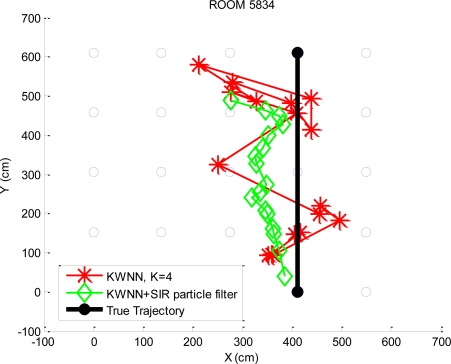
The dynamic positioning results by the KWNN algorithm (*K* = 4).

**Figure 14.**
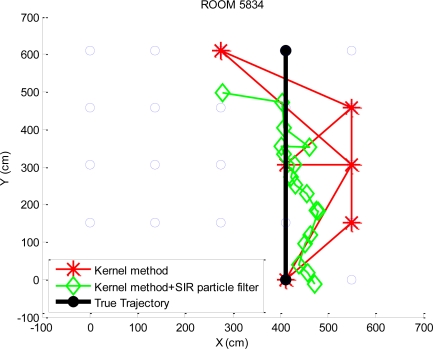
The dynamic positioning results by the kernel method.

The dynamic experimental results of the KWNN algorithm and the KWNN algorithm with the SIR particle filter of *K* = 4 are also shown in Figure 13. Table 2 is the standard deviations of positioning errors by applying the kernel method and the kernel method with the SIR particle filter. The dynamic positioning results of the kernel method and the kernel method with the SIR particle filter are illustrated in Figure 14. The positioning error is defined as the difference between the positioning result and the true trajectory. It is obvious that the standard deviations of the positioning errors by applying the SIR particle filter are smaller. It means that the SIR particle filter can enhance the positioning performance as the object is in motion. From Table 2, it also shows that the standard deviations of the positioning errors are similar while selecting more candidates for the kernel method without the SIR particle filter. This experiment result is in accordance with the static experiment analysis in Figure 11.

**Figure 15.**
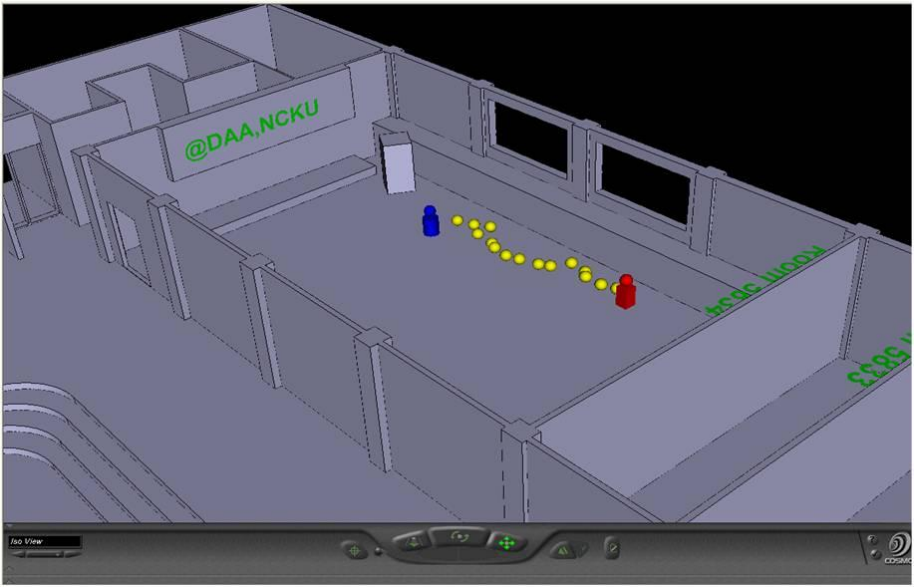
Integration of the indoor positioning results with the 3D GIS.

Finally, the indoor positioning results are integrated with the developed 3D GIS to show the user position in the 3D map. The integration result is shown in Figure 15. In Figure 15, the blue man represents the start position, the yellow balls are the user’s trajectory, and the red man is the end position. The VRML player used in this paper is the Cosmo player, and the user can choose other VRML player as well.

## 5. Conclusions

In this paper several matching fingerprinting method algorithms are investigated to analyze their indoor positioning results, and the matching algorithms used in this paper included the nearest neighbor (NN) algorithm, the K-weighted neatest neighbor (KWNN) algorithm and the probabilistic approach based on the kernel method. The experiment results of this paper indicated that the KWNN algorithm with parameter *K* = 3 or 4 gave the best indoor positioning result. In addition, the sampling importance resampling (SIR) particle filter is used in this paper to enhance the indoor positioning performance. With the assistance of the SIR particle filter, the estimated indoor trajectories became smoother. As shown in the experiment results, an indoor positioning system is successfully developed in this paper, and more importantly, this paper proposes a practical and low cost procedure to construct a three-dimensional (3D) indoor geographic information system (GIS) based on the computer-aided design (CAD) software and the virtual reality markup language (VRML) technique. The developed 3D indoor GIS of this paper has high flexibilities to be integrated with the indoor positioning system, and the user can choose many available VRML players to use the developed system to gain local information based on their locations. Finally, the Department of Aeronautics and Astronautics building at National Cheng Kung University was used as an example to successfully demonstrate the location based service (LBS) test bed with wireless sensor network (WSN).

## Figures and Tables

**Table 1.** Standard deviations of positioning errors of the dynamic test using the KWNN algorithm and the KWNN algorithm with the SIR particle filter.

***K***	**1**	**2**	**3**	**4**
**Algorithm**				
KWNN (cm)	99.86	51.44	38.36	58.33
KWNN + SIR particle filter (cm)	24.22	33.30	22.69	26.53

**Table 2.** Standard deviations of positioning errors of the dynamic test using the kernel method and the kernel method with the SIR particle filter.

**Candidate**	**1**	**2**	**3**	**4**
**Algorithm**				
Kernel method (cm)	64.46	63.97	63.89	63.89
Kernel method + SIR particle filter (cm)	32.89	32.18	33.99	29.68

## References

[1] Rao B., Minakakis L. (2003). Evolution of mobile location-based services. J. Commun. ACM.

[2] van Lammeren R., Goossen M., Roncken P. (2009). Sensing landscape history with an interactive location based service. Sensors.

[3] Lloret J., Tomas J., Garcia M., Canovas A. (2009). A hybrid stochastic approach for self-location of wireless sensors in indoor environments. Sensors.

[4] Ni L.M., Liu Y., Lau Y.C., Patil A.P. (2004). Landmarc: Indoor location sensing using active RFID. Wirel. Netw.

[5] Tan K.M., Law C.L. GPS and UWB integration for indoor positioning.

[6] Goldsmith A. (2005). Wireless Communications.

[7] Cubic I., Begusic D., Mandic T. Client based wireless LAN indoor positioning.

[8] LeMaster E.A., Rock S.M. (2003). A local-area GPS pseudolite-based navigation system for mars rovers. J. Auton. Robots.

[9] Qiu D., Lorenzo D.D., Lo S., Boneh D., Enge P. Physical pseudo random function in radio frequency sources for security.

[10] Wang H., Lenz H., Szabo A., Bamberger J., Hanebeck U.D. WLAN-based pedestrian tracking using particle filters and low-cost MEMS sensors.

[11] Roos T., Myllymäki P., Tirri H., Misikangas P., Sievänen J. (2002). A probabilistic approach to WLAN user location estimation. Int. J. Wireless Inf. Networks.

[12] Li B., Wang Y., Lee H.K., Dempster A., Rizos C. (2005). Method for yielding a database of location fingerprints in WLAN. IEE Proc.-Commun.

[13] Arulampalam M.S., Maskell S., Gordon N., Clapp T. (2002). A tutorial on particle filters for online nonlinear/non-gaussian bayesian tracking. IEEE Trans. Signal Process.

[14] Carey R., Bell G. (1997). The Annotated VRML 2.0 Reference Manual.

[15] Bahl P., Padmanabhan V.N. RADAR: An in-building RF-based user location and tracking system.

[16] Ristic B., Arulampalam S., Gordon N. (2004). Beyond the Kalman Filter: Particle Filters for Tracking Applications.

[17] Widyawan M.K., Pesch D. Influence of predicted and measured fingerprint on the accuracy of RSSI-based indoor location systems.

